# Effects of Hybridized Organically Modified Montmorillonite and Cellulose Nanocrystals on Rheological Properties and Thermal Stability of K-Carrageenan Bio-Nanocomposite

**DOI:** 10.3390/nano9111547

**Published:** 2019-10-31

**Authors:** Siti Zarina Zakuwan, Ishak Ahmad

**Affiliations:** Centre for Advanced Materials and Renewable Resources, Faculty of Science and Technology, Universiti Kebangsaan Malaysia, Bangi Selangor 43600, Malaysia

**Keywords:** kappa-carrageenan, synergistic nanoparticles, hybrid composite, rheological properties, thermal stability, nanocellulose, nanoclay

## Abstract

Herein, hybrid k-carrageenan bio-nanocomposite films were fabricated by using two types of nanofillers, organically modified montmorillonite (OMMT), and cellulose nanocrystals (CNCs). Hybrid bio-nanocomposite films were made by casting techniques employing 4 wt% of CNCs, OMMT, and hybridized CNCs/OMMT in a 1:1 ratio. The rheological and morphological properties and thermal stability of all composites were investigated using rotational rheometry, thermogravimetry analysis, differential scanning calorimetry, field emission scanning electron microscopy, and transmission electron microscopy (TEM). The results showed that the hybrid CNC/OMMT bio-nanocomposite exhibited significantly improved properties as compared to those for the bio-nanocomposites with single fillers due to the nanosize and homogenous nanofiller dispersion in the matrix. Rheological analysis of the hybrid bio-nanocomposite showed higher dynamic shear storage modulus and complex viscosity values when compared to those for the bio-nanocomposite with individual fillers. The TEM analysis of the hybridized CNC/OMMT bio-nanocomposite revealed that more particles were packed together in the CNC network, which restricted the matrix mobility. The heat resistance and thermal stability bio-nanocomposite k-carrageenan film enhanced rapidly with the addition of hybridized CNCs/OMMT to 275 °C. The hybridized CNCs/OMMT exhibited synergistic effects due to the good affinity through interfacial interactions, resulting in the improvement of the material properties.

## 1. Introduction

Biopolymers formulated with reinforcement materials such as natural fibers, nanoparticles, and synthetic fibers are currently being developed because of their unique properties to reduce the usage of synthetic plastics, particularly for short-term packaging and disposable materials [1,2]. K-carrageenan belongs to a class of renewable materials with abundant resources obtained by the extraction of red seaweeds (*Eucheuma cottonii*) and consisting of repeating 4-linked 3,6-anhydro-D galactose and 4-linked β-D-galactose-4-sulphate units with anionic sulfate groups (OSO_3_^−^) disaccharide units [3,4]. The excellent film-forming ability of k-carrageenan has significant potential for the preparation of composite films because of the linear chain of partially sulfated galactan molecules with one negative charge per disaccharide unit. This polymer is non-toxic, biodegradable, and is widely used in the healthcare and cosmetic industries and food processing due to the hydrophilic and ionic properties, which can be exploited in various applications [5,6]. The ideal packaging materials must have excellent mechanical properties, good thermal stability, and should be biodegradable [7,8,9]. Bio-nanocomposite films with inorganic molecules of different structures and morphologies with at least one phase shows that dimensions of less than 100 nm had been used for improving film properties and the application of biodegradable materials.

Nanocellulose that can be extracted from abundant natural fiber exhibits properties such as relatively large surface areas, low density, high specific stiffness, high strength, and easy processability [10,11,12]. Cellulose nanocrystals (CNCs) are versatile and can be used in various applications such as reinforcing fillers, rheological modifiers, pharmaceutical carriers, biomedical implants, and substrates for electronic components [13]. El-Miri et al. [14] reported that the increase of tensile strength and elastic modulus with the addition of CNCs in the carboxymethyl cellulose/starch nanocomposite. CNCs have also been reported to improve the tensile strength of alginate biopolymer [15,16].

Nanocomposites based on polysaccharide and nanoclay reinforcements are widely used across various research fields as clay minerals are cheaper, and biodegradable nano additives used to enhance the properties of polymers materials are known as polymer–clay nanocomposites [1,3,17,18,19]. Organomontmorillonite reinforced biopolymers promise to be an attractive replacement for the invention of environmentally friendly products for the elimination of pollutants [20]. The addition of organically modified montmorillonite (OMMT) has already reported to enhance the mechanical properties of polyurethane adhesive. The shearing strength, fracture tensile strength, and peel strength of the polyurethane with the addition of 4 wt% OMMT were superior [21]. Luo et al. [22] reported the enhancement of the thermal stability of the polylactic acid/OMMT nanocomposite due to better compatibility with the matrix phase. 

In prior studies, k-carrageenan bio-nanocomposite materials were studied with a focus on the effects of the CNC and OMMT on the mechanical properties to determine the optimum nanoparticle loadings. Morphological studies show the well distribution of CNC and OMMT nanoparticles in the matrix. Hybridized CNC/OMMT nanofillers exhibit synergistic effects resulting in excellent mechanical properties, good water resistance, with decreasing absorption of water of the bio-nanocomposite in contrast to the bio-nanocomposites with single fillers [23]. Previous study using CNCs as reinforcing materials in starch biopolymers has shown that the storage modulus increased with the addition of CNC due to good CNC–matrix interaction and stiffness properties of CNC [24]. The matrix polymer and reinforcement nanoparticles are the two major constituents of a composite material which synergistically increase the stability, thermal expansion, and viscosity of the composite [25]. However, the hybridization of CNC/OMMT to a biopolymer matrix and the resulting research rheological behavior and thermal stability of this novel hybrid bio-nanocomposite have not been reported previously.

The ultimate aims of this current work are to show how the hybridization of different organic and inorganic nanofillers affect the rheological behavior and thermal stability properties, and to extensively investigate the entanglement of the nanoparticles inside the matrix for applications depending on the location distribution of the nanofillers. Rheological properties of the materials are important during polymerization as well as downstream processing of the product (e.g., extraction, injection molding, and fiber spinning). The measurement of the viscosity of the bio-nanocomposite can provide direct evidence for changes in the product quality due to the destabilization in a polymer [26].

## 2. Materials and Methods 

### 2.1. Reagents and Materials

Kenaf fiber was purchased from Kenaf Fiber Malaysia Sdn. Bhd. (Kelantan, Malaysia) and k-carrageenan was provided by TACARA Sdn. Bhd. (Sabah, Malaysia). Methyl dihydroxyethyl hydrogenated tallow ammonium (OMMT 25–30 wt%) was purchased from AMCOL International Corp. (Nanocor^®^; Hoffman Estates, IL, USA). Sulfuric acid (98%), Glycerol (99.5%), glacial acetic acid (99.5%), sodium chlorite (80%), and sodium hydroxide (99%), were obtained from SYSTERM-chemAR (Selangor, Malaysia) and Sigma-Aldrich (St. Louis, MO, USA). Membrane tubes (1 inch × 10 feet; Carolina Dialysis Tubing, Carolina Biological Supply Company, Burlington, NC, USA) were used for dialysis. 

### 2.2. Preparation of Cellulose Nanocrystals 

CNCs were extracted from kenaf fiber via the sulfuric acid hydrolysis process as described by Kargarzadeh et al. [23]. Cellulose was produced from kenaf fiber by alkaline treatment for 3 h at 80 °C. The bleaching process was performed for 4 h at 80 °C, followed by hydrolysis with sulfuric acid (65%). CNCs were then washed with distilled water to remove excess sulfuric acid by using the centrifugation process. 

### 2.3. Preparation of Bio-Nanocomposite Films

The fabrication of k-carrageenan–cellulose nanocrystal (k-carr–CNC), k-carrageenan–organically modified montmorillonite (k-carr–OMMT), and hybrid k-carrageenan–cellulose nanocrystal/organically modified montmorillonite (k-carr–CNCs/OMMT) bio-composite films was accomplished by the solution casting technique. K-carrageenan was first mixed with the 4% (w/w) nanofillers in distilled water. The suspension was heated in the range of 70–90 °C. The suspension was continuously stirred for another 30 min after the heating was removed. The thickness of the bio-nanocomposite film was controlled using a casting knife before being dried at room temperature to obtain the final film thickness in the range of 30–50 µm.

### 2.4. Morphological Analysis

The morphological observation of the fibers after each treatment was examined via field-emission scanning electron microscopy (FESEM) at a magnification of 1.00 K× and 15.00 K× (Philips XL-3, Amsterdam, The Netherlands). The morphology of the CNCs particles and bio-nanocomposite film in the water medium was analyzed by using transmission electron microscope (Philips CM30, Amsterdam, The Netherlands), as described by Zakuwan and Ahmad [23].

### 2.5. Rheological Analysis 

The rheological behavior of the polymers was carried out using a dynamic rotational rheometer (Physica MRC 301, Anton Paar GmbH, Graz, Austria) as described by Noranizan et al. [27]. The storage and loss moduli and complex viscosities were measured.

### 2.6. Thermogravimetric Analysis (TGA)

The thermal stability and degradation studies of the bio-nanocomposite films were performed using a thermogravimetric analyzer (Mettler Toledo, TGA/SDTA 851-E, Columbus, Ohio, USA) under a nitrogen atmosphere. Each sample was heated from 25 °C to 600 °C at 10 °C·min^−1^. 

### 2.7. Differential Scanning Calorimetry (DSC) 

Thermal stability characteristics of the k-carrageenan bio-nanocomposite were determined using a differential scanning calorimeter (Mettler Toledo-DSC 822e, Columbus, Ohio, USA). The heating processed up to 200 °C at a rate of 10 °C·min^−1^. 

## 3. Results and Discussion 

### 3.1. Morphological Analysis

#### 3.1.1. Fiber Morphology

**Figure 1:**
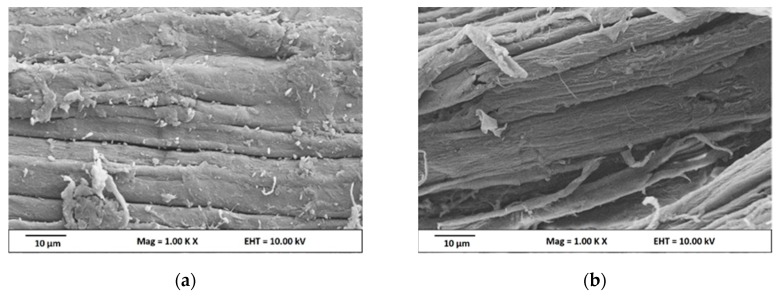

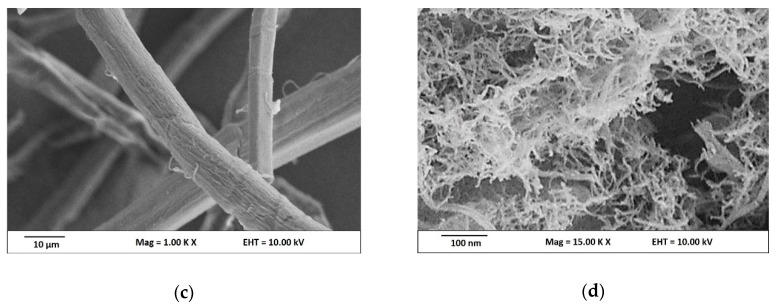
Scanning Electron Microscopy images of (**a**) raw kenaf, (**b**) alkali kenaf, (**c**) bleach kenaf, and (**d**) nanocellulose kenaf.

**Figure 2:**
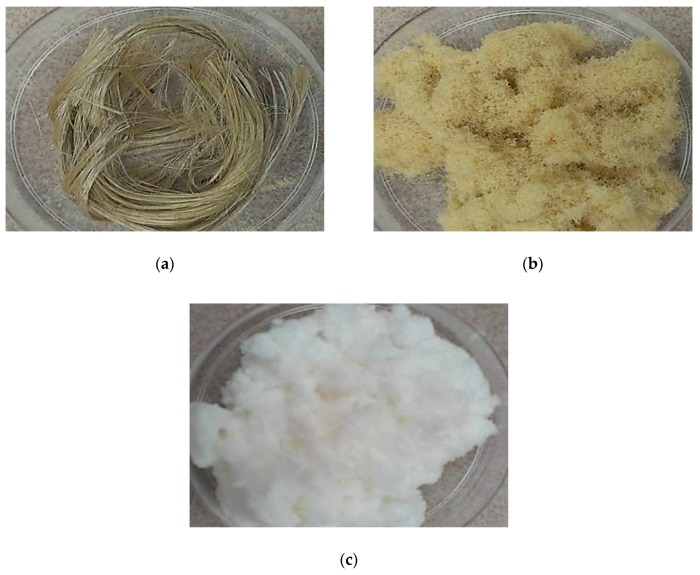
Photograph of (**a**) raw kenaf, (**b**) alkali kenaf, (**c**) bleach kenaf.

The scanning electron microscopy (SEM) micrograph of the kenaf raw fiber after different chemical treatments are shown in Figure 1. The chemical treatment involved the use of a combination of alkaline treatment, bleaching, and acid hydrolysis to remove hemicellulose and lignin, affording microsize yields of pure cellulose from which nanocellulose particles were successfully extracted. Kenaf raw fiber (Figure 1a) is composed of individual large fiber bundles with a compact surface structure coated with impurities. The presence of lignin and hemicelluloses around the fibrils can be clearly observed on the surface. However, after treatment with the alkaline solution, the kenaf fibers (Figure 1b) showed a surface with rough topography because of the elimination of some of the lignin and hemicellulose, impurities, and oily substances from the fiber. The alkalization process with sodium hydroxide, which is well-known for removing the undesirable material and amorphous part from the surface, results in the partial defibrillation of the fibers [28]. During the alkalization at high temperature, lignin is partially depolymerization and most of the hemicellulose is hydrolyzed with an increase in the internal surface area [29]. After bleaching (Figure 1c), the kenaf fiber showed a smooth and clean surface with microsize dimensions due to the defibrillation of fibers. Alkaline treatment and bleaching are common pre-processing techniques used on natural fibers to remove lignin/hemicellulose and modify the fiber surface to reduce the surface tension and improve interfacial adhesion between the natural fiber and polymer matrix [30]. As shown in Figure 1d, the CNC production was clearly observed after acid hydrolysis. This chemical treatment led to a significant decrease in the fibril length because during hydrolysis, the acid dissociates the amorphous part of the microfibrils, leaving the highly crystalline region intact. Accordingly, the hydronium ions can penetrate into the amorphous domains and stimulate the hydrolytic cleavage of the glycosidic bonds, releasing individual nanosized cellulose structures that contain highly crystalline domains [31]. Figure 2 shows the optical micrograph of the kenaf fiber after the alkali and bleaching treatments. The color of the raw kenaf fiber became lighter after alkali treatment and completely white after bleaching treatment. 

**Figure 3:**
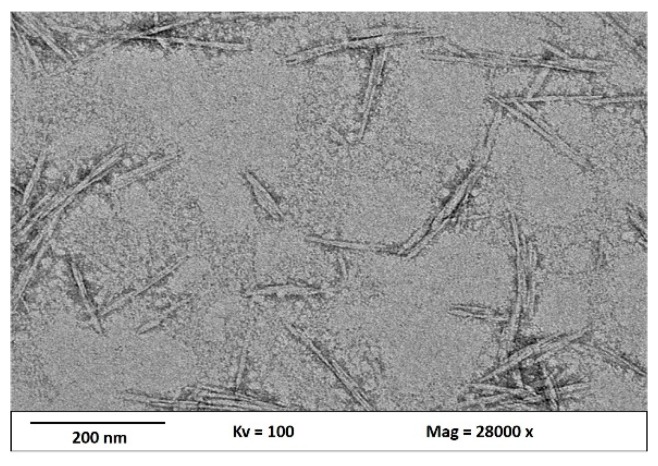
Transmission Electron Microscope images of hydrolyzed kenaf at a magnification of 28,000×.

The morphologies and sizes of the obtained CNCs were determined using TEM. TEM images of the CNCs produced after the acid hydrolysis in water are shown in Figure 3. It can be seen that the amorphous domain was dissolved by acid hydrolysis, leaving the highly crystalline domain intact. The TEM micrograph of CNCs shows that the nanoparticles exhibit needle-like shapes, which is consistent with the previously reported results [32,33,34]. CNCs have a dimension of 12–15 nm in diameter and 101–260 nm in length. The CNCs have a high aspect ratio, which plays a crucial role in their reinforcement capability [35,36]. Some lateral agglomeration can be clearly observed due to hydrogen bonding of the CNC particles [37]. Table 1 presents the kenaf fiber dimension after different stages of chemical treatments.

#### 3.1.2. Bio-Nanocomposite Morphology

**Figure 4:**
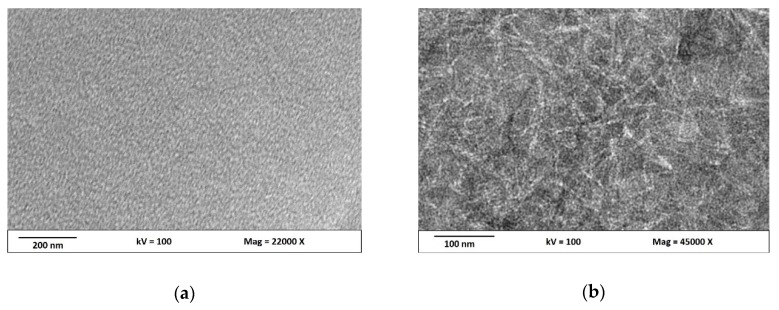
Transmission Electron Microscope images of the (**a**) k-carrageenan matrix and (**b**) hybrid bio-nanocomposite (CNCs/OMMT) with 4-wt% filler loading.

The morphology of the hybrid CNCs/OMMT bio-nanocomposite films were analyzed using TEM. Figure 4a shows the TEM image of the k-carrageenan matrix film at a magnification of 22,000×. A relatively homogeneous and smooth film surface was observed. Figure 4b shows the TEM image of the hybrid bio-nanocomposite at a magnification of 45,000×. It can be seen that both CNCs and OMMT were homogeneously dispersed within the polymer matrix. The CNCs particles formed a network in the matrix together with the OMMT particles. The distributions of the OMMT and CNC particles in the matrix showed intercalated OMMT with some layered clay particles with different sizes and platelet orientations. Some particles were ordered in a multilayer nanostructure, while others were the dispersed OMMT particles distributed in the matrix. Different dimensions of the CNC particles in the network were observed and the OMMT particles were incorporated in the matrix space. This shows the strong interfacial interaction due to the entanglement of the CNCs and OMMT nanofillers in the hybrid k-carr-CNCs/OMMT system because of a high specific area, which increases surface interaction and leads to synergistic effects.

### 3.2. Rheological Analysis 

**Figure 5:**
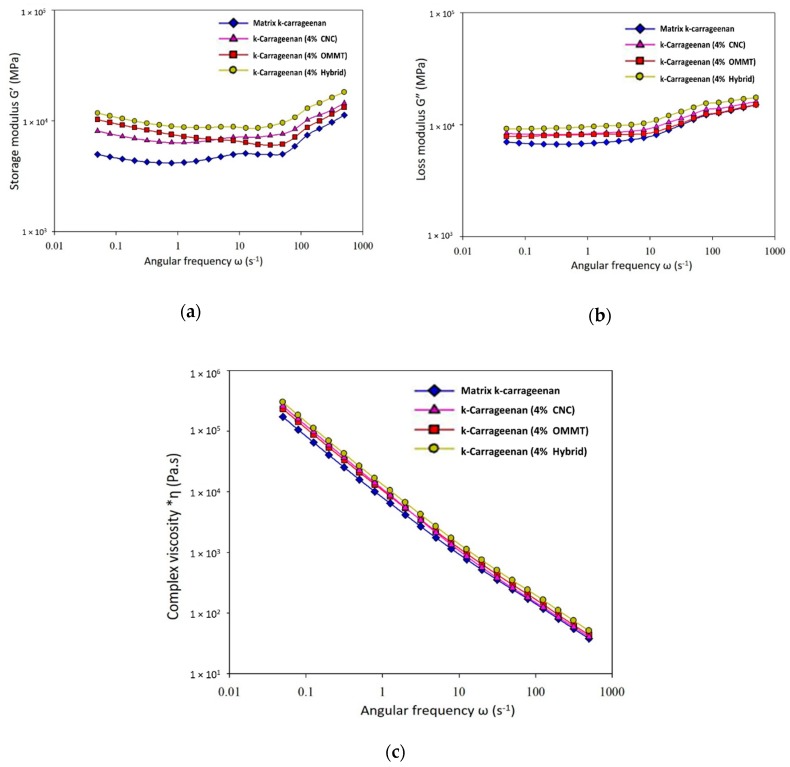
Rheological behavior: (**a**) storage moduli (G’), (**b**) loss moduli (G”), and (**c**) complex viscosities (η*) of the bio-nanocomposite films.

A rheological study of the bio-nanocomposites showed high complex viscosities and dynamic shear storage moduli for the CNCs, OMMT, and hybridized CNCs/OMMT. This study is useful for understanding the relationship between the nanofillers. Figure 5 shows that the addition of nanofillers as reinforcement materials in the polymer matrix affects the values of the storage modulus (G’; Figure 5a), loss modulus (G”; Figure 5b), and complex viscosity (η*; Figure 5c). The results for the bio-composites with 4% filler loadings of CNCs, OMMT, and hybrid CNCs/OMMT are shown in these figures. Different values for G’ and G” correspond to the different types of fillers used in the polymer matrix. Increases in G’ and G” values with increasing frequencies were observed for all bio-nanocomposite samples. The addition of CNCs, OMMT, and hybridized CNCs/OMMT (Figure 5a,b) results in a Newtonian behavior at low shear rates. As shown in Figure 5a, the bio-nanocomposite with the addition of CNC had a higher storage modulus, G’, at a higher frequency than that for the bio-nanocomposite with OMMT. Elastic modulus is related to the surface interaction [38]. CNCs with the same chemical structure and polarity as the polymer matrix showed better compatibility, leading to an enhanced matrix–filler interaction in the composite film. The CNCs in the polymer matrix formed a network (Figure 4), which enhanced the elastic properties of the composite, particularly at high frequency. At the early stage, the k-carrageenan/OMMT bio-nanocomposite showed a high G’ value under shear flow due to the high energy needed to overcome the strong interaction between the k-carrageenan matrix and OMMT. This shows that strong ionic interactions develop between the matrix and OMMT. High anionic charges (–OSO_3_^−^) on k-carrageenan enable its interaction with the inorganic cations, with polymer chain intercalation [23]. A decrease in the G’ value of k-carrageenan/OMMT at high frequency was due to the intercalation of the OMMT particle without full exfoliation so that less energy is used to rearrange the OMMT particles. The TEM image clearly shows the intercalated OMMT in the polymer matrix. The bio-nanocomposite sample with hybridized CNCs/OMMT showed the highest G’ value due to the network formation and homogeneously dispersed CNCs/OMMT in the matrix as well as strong filler–matrix interaction. The stiffness of the bio-nanocomposite improves with the addition of CNCs/OMMT due to the higher stiffness of both nanomaterials. Thus, the hybrid bio-nanocomposite possesses high strength with better flexibility, which is related to the restricted polymer chain movement and high energy requirement [39]. Figure 5b shows no significant differences in the G” values for the composites with CNCs and OMMT. However, the bio-composite with hybridized CNCs/OMMT showed a slightly higher G” value with an increase in frequency when compared to other samples. 

The variation in the complex viscosity values for the neat matrix and bio-nanocomposite films is shown in Figure 5c. The complex viscosity (η*) of bio-nanocomposite 4% fillers was higher than the matrix without nanofillers and the value decreased with an increase in frequency. The bio-composite with hybridized CNCs/OMMT had a higher η* value than the bio-nanocomposites with the single fillers. All bio-nanocomposite samples had higher viscosities when compared to that for a neat polymer. The incorporation of both filler CNCs and OMMT in the matrix k-carrageenan caused the viscosity of the matrix to increase due to the enhancement of the interaction between k-carr/CNCs and k-carr/OMMT. As shown in Figure 5c, the viscosity of mixing increased, particularly at a low shear rate. This behavior is associated with the formation of a network filler structure, leading to the enhancement of the matrix–filler interaction, such that a high resistance is observed under a shear flow. Increasing the shear rate, the intermolecular junction decays at a faster rate than their renewal rate, resulting in a decrease in viscosity [14]. A 4% filler loading causes a higher entanglement of the chains and particles, and results in a gradual increase in the η* values when compared with the k-carrageenan matrix without nanofillers [40]. Nanoparticles form a network based on the interactions between the particles and their dimensions (aspect ratio). A similar trend was previously reported in the literature where the bio-nanocomposites showed shear thinning behavior in the complete range of shear rate values, which attributed to the formation of CNC network structures in the neat matrix [41]. The hybrid bio-nanocomposite rigidity improved and a slight increase in the shear viscosity was observed with the incorporation of hybrid CNCs/OMMT with better particle distribution. These results confirm that a better interaction between both CNCs and OMMT in the polymer matrix were observed in a hybrid composite due to the addition of different types of fillers. The phenomena were predominant due to better dispersion of nanoparticles with different dimensions. An increase in viscosity occurred, and the composite system interfered with the normal flow of the polymer and inhibited the movement of the mobility of the chain segments. 

### 3.3. Thermogravimetric Analysis

**Figure 6:**
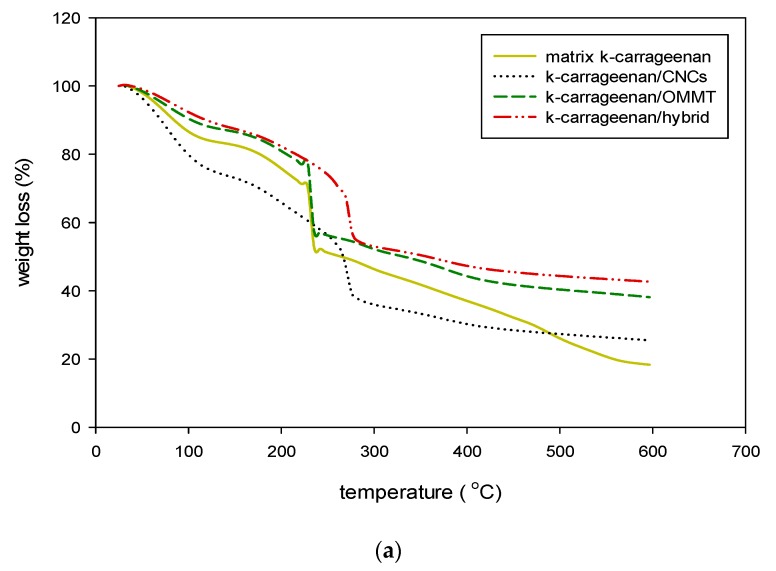

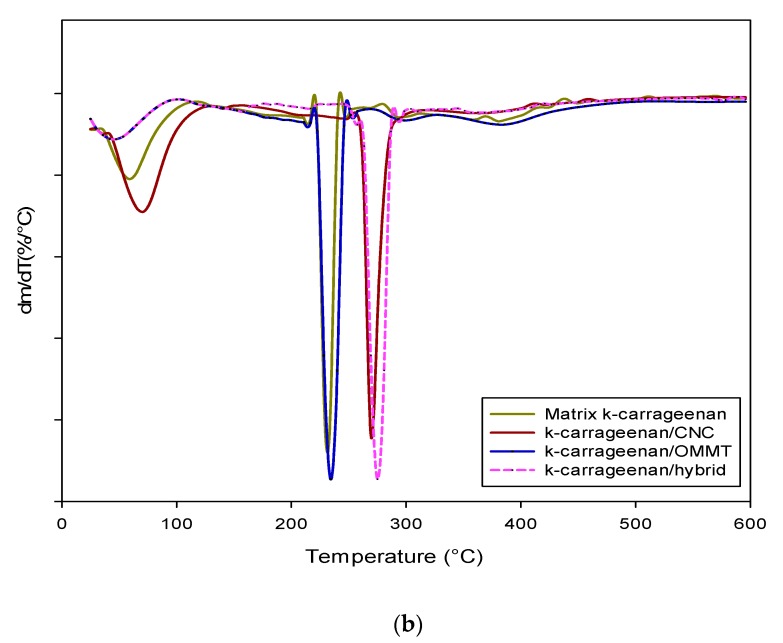
Thermogravimetric analysis (TGA) curve of the k-carrageenan matrix film and k-carrageenan bio-nanocomposites with 4-wt% filler loading (**a**), Derivative Thermogravimetry (DTG) curves of the k-carrageenan matrix film and k-carrageenan bio-nanocomposites with 4-wt% filler loading (**b**).

The thermal stability of the k-carrageenan matrix and bio-nanocomposite with the addition of CNCs, OMMT, and hybridization was investigated by using TGA. Figure 6 shows the TGA results where the samples with different types of filler loadings (4 wt%) were analyzed and the data were compared with that of the pure matrix. Table 2 summarizes the degradation results for all samples. Thermal degradation of the bio-nanocomposites and matrix films exhibited early-stage degradation from 50 °C to 150 °C. The initial degradation occurred due to the removal of moisture content in the material and the water evaporation process. The first-stage degradation due to the absorption of water by the materials was high because water could penetrate via the interactions of the hydroxyl groups in the glucosyl units along with the chain and hydroxyl groups of the plasticizer [35,42]. The decline in the mass losses of the neat k-carrageenan matrix and bio-nanocomposites at 125–230 °C refers to the thermal degradation of glycerol and polysaccharides [43].

Further degradation of the k-carrageenan matrix film occurred at approximately 228–236 °C. The addition of CNCs to the k-carrageenan matrix increased the decomposition temperature to approximately 264–278 °C at 4-wt% filler loading. The maximum degradation occurred at 270 °C. Furthermore, the addition of CNCs to k-carrageenan significantly increased the mass loss because the CNCs were less thermally stable compared to OMMT. Higher thermal decomposition with the incorporation of CNC due to high crystallinity, and had a higher aspect ratio of the CNC that was well dispersed within the matrix. This result shows that CNCs interacted with the matrix k-carrageenan and enhanced the thermal degradation of the corresponding bio-nanocomposites to a greater extent. Meanwhile, a drastically degradation rate due to the presence of negative sulfate groups (SO_4_^2−^) [44]. The addition of OMMT to k-carrageenan did not show significant changes in the thermal degradation of the bio-nanocomposite from 227–236 °C, with a maximum degradation at 233 °C. Roy and Rhim [45] showed similar results, where the addition of melanin nanofillers did not show any significant differences in comparison with the neat matrix. In contrast, the k-carr/OMMT bio-nanocomposite residue increased (38.11%) in comparison to that of the k-carrageenan matrix, mainly attributed to the well dispersion of thermally stable OMMT in the bio-nanocomposite. Therefore, the decomposition that occurred at 233 °C was due to the degradation of the k-carrageenan matrix and increased slightly, which was caused by the addition of OMMT. Thus, the final residue showed a higher OMMT increase in the crystallinity of the k-carr/OMMT bio-nanocomposite, while there was an increase in the thermal stability and resistance degradation of the biopolymer. The degradation of the hybrid k-carr/CNC/OMMT bio-nanocomposite was observed at 268–284 °C, where the main degradation peak was at approximately 275 °C, with a residue of 42.65%. The addition of hybrid CNCs/OMMT showed significant changes in the thermal stability. For the bio-nanocomposite prepared with hybridized CNCs/OMMT, a trend similar to that for the k-carr/CNC bio-nanocomposite was observed. All bio-nanocomposite samples showed better thermal degradation than the k-carrageenan matrix. Moreover, the higher thermal stability of the hybrid k-carrageenan with the CNCs/OMMT than those of the individual nano-reinforcement bio-nanocomposites because of the physical interaction from hydrogen bonding and the ionic interaction of OMMT and k-carrageenan through cationic exchanges as well as better interfacial interaction because of the high aspect ratio of OMMT. Figure 6b shows the thermal analysis decomposition pattern of the matrix k-carrageenan and bio-nanocomposite with 4% filler loading during heating. 

### 3.4. Differential Thermal Analysis 

**Figure 7:**
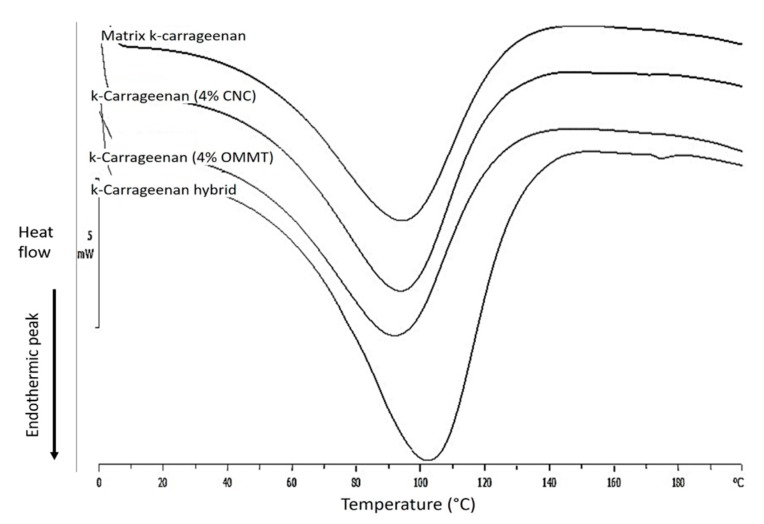
DSC thermograms of neat matrix k-carrageenan and bio-nanocomposite k-carrageenan with a 4-wt% filler loading.

Differential scanning calorimetry (DSC) data (Figure 7) for the bio-nanocomposite films showed endothermic peaks that corresponded to the transition over a broad temperature range. Similar to the results reported in prior studies, the k-carrageenan pure matrix gel and k-carrageenan with other polymers also exhibited one endothermic peak throughout the melting process in the DSC spectrum [46]. The DSC spectrum shows the transition temperature, where the difference between the onset temperature, T_o_, and the peak temperature, T_p_, is important to determine whether a material is close enough to lose its structure during the heating process [47]. All films presented in Figure 7 exhibited endothermic peaks in the range of 54–129 °C. A neat k-carrageenan matrix film exhibited a broad endothermic peak at 59–123 °C with a T_p_ of 94 °C. A factor that influences the sealing strength is the hydroxyl groups in the plasticizers. Endothermic peaks for k-carr/CNCs were observed at 54–120 °C with a T_p_ of 94 °C. This proves that k-carr/CNCs show a higher transition temperature when compared to the neat matrix, with the lowest onset temperature and afforded better seal strength. The K-carr/OMMT bio-nanocomposite exhibited a transition temperature in the range of 54–115 °C with a T_p_ of 93 °C, which was lower than that of the neat matrix. Based on the TGA results, k-carr/OMMT only affords an increase in thermal stability (Figure 6). The DSC spectrum of the hybrid k-carr/CNC/OMMT bio-nanocomposite showed a shift in the endothermic peak to a higher temperature. K-carr/CNCs/OMMT showed endothermic peaks at T_p_ of 103 °C in the region between 64–129 °C. The changes that occurred at the transition temperature can be attributed to high rigidity and low mobility of the intermolecular chains during heating. The addition of CNCs and OMMT showed synergistic effects on the thermal properties because of better interfacial interaction between the nanoparticles with a high aspect ratio and matrix, which afforded a superior material with high seal strength and high degradation temperature when compared to the bio-nanocomposite films with single fillers. Table 3 represents the values of endothermic peaks for the bio-nanocomposite films k-carrageenan with the 4-wt% filler loading.

## 4. Conclusions

Cellulose from kenaf and CNCs with average diameters and lengths of 12–15 nm and 101–260 nm, respectively, were obtained using alkaline treatment, bleaching, and acid hydrolysis. K-carrageenan bio-nanocomposite films reinforced with 4-wt% CNCs and OMMT were prepared via a casting technique. The hybrid bio-nanocomposite was prepared with the addition of 4-wt% CNCs/OMMT (1:1). Different types of nanofillers enhanced the thermal stabilities and rheological properties of the bio-nanocomposites. Hybrid bio-nanocomposite films showed a significant enhancement of various properties when compared with those for the k-carrageenan with CNC and k-carrageenan with OMMT bio-nanocomposite. Bio-nanocomposite films with CNCs/OMMT showed better rheological characteristics among the three formulations due to the entanglement of CNCs/OMMT inside the matrix phases, and the development of a network filler structure leading to the enhanced filler–matrix interactions. TEM data revealed that the OMMT particles were packed together in the CNC network, which restricted the matrix mobility. The hybrid bio-nanocomposite films exhibited synergistic effects with higher thermal degradation temperature when compared to the bio-nanocomposite films with single fillers. This improvement was associated with the homogeneous and well dispersed CNCs and OMMT, high aspect ratio for different dimensions, and the interfacial and ionic interactions between CNCs and k-carrageenan functional groups. It can be concluded from this study that a uniform mixing of the hybridization of CNC/OMMT with different polarities like CNCs and OMMT can increase the rheological behavior and that the thermal stability properties have the ability to make a fully biodegradable nanocomposite, and hence present a wide range of potential in packaging and coating applications. 

## Figures and Tables

**Table 1:** Fiber dimensions after different stages of chemical treatments.

Sample	Fiber Diameter
Raw kenaf	>100 μm
Alkali-treated kenaf	20–90 μm
Bleach-treated kenaf	10–14 μm
Hydrolyzed kenaf (CNC)	12–15 nm

**Table 2:** TGA maxima, thermogravimetric analysis data, corresponding peak, and residue values for the bio-nanocomposite films.

Sample	Onset (°C)	Peak (°C)	Endset (°C)	Residue (%)
k-carrageenan matrix	228	230	236	18.32%
k-carrageenan (4% CNCs)	264	270	278	25.49%
k-carrageenan (4% OMMT)	227	233	236	38.11%
k-Carrageenan (hybrid)	268	275	284	42.65%

**Table 3:** Values of the endothermic peaks for the bio-nanocomposite films with k-carrageenan with 4-wt% filler loading.

Sample	Onset (°C)	Peak (°C)	Endset (°C)	Integral (mJ)
k-Carrageenan matrix	59.34	94.67	123.38	−1440.93
k-Carrageenan (4% CNCs)	54.98	94.50	120.86	−1445.04
k-Carrageenan (4% OMMT)	54.00	93.67	115.48	−1337.71
k-Carrageenan (hybrid)	64.80	103.00	129.29	−2177.67

## References

[1] Shojaee-Aliabadi S., Mohammadifar M.A., Hosseini H., Mohammadi A., Ghasemlou M.S., Hosseinia M., Haghshenas M., Khaksara R. (2014). Characterization of nanobiocomposite κ-carrageenan film with Zataria multiflora essential oil and nanoclay. Int. J. Biol. Macromol..

[2] Gonzalez-Gutierrez J., Partal P., Gallegos M., Gallegos. C. (2011). Effect of processing on the viscoelastic, tensile and optical properties of albumen/starch-based bioplastics. Carbohydr. Polym..

[3] Liu J., Zhan X., Wan J., Wang Y., Wang C. (2015). Review for carrageenan-based pharmaceutical biomaterials: Favourable physical features versus adverse biological effects. Carbohydr. Polym..

[4] Tytgata L., Vagenendea M., Declercqc H., Martinsd J.C., Thienpontb H., Ottevaereb H., Dubruela P., Van Vlierberghea S. (2018). Synergistic effect of κ-carrageenan and gelatin blends towards adipose tissue engineering. Carbohydr. Polym..

[5] Rhim J.W., Wang L.F. (2013). Mechanical and water barrier properties of agar/κ-carrageenan/konjac/ glucomannan ternary blend biohydrogel films. Carbohydr. Polym..

[6] Oun A.A., Rhim J.W. (2017). Carrageenan-based hydrogels and films: Effect of ZnO and CuO nanoparticles on the physical, mechanical, and antimicrobial properties. Food Hydrocoll.

[7] Tavassoli-Kafrani E., Shekarchizadeh H., Masoudpour-Behabadi M. (2016). Development of edible films and coatings from alginates and carrageenans. Carbohydr. Polym..

[8] Wang Y., Yuan C., Cui B., Liu Y. (2018). Influence of cations on texture, compressive elastic modulus, sol-gel transition and freeze-thaw properties of kappa-carrageenan gel. Carbohydr. Polym..

[9] Wurm F., Pham T., Bechtold T. (2019). Modelling of phase separation of alginate-carrageenan gels based on rheology. Food Hydrocoll..

[10] Sapkota J., Gooneie A., Shirole A., Martinez Garcia J.C. (2017). A refined model for the mechanical properties of polymer composites with nanorods having different length distributions. J. Appl. Polym. Sci..

[11] Sapkota J., Martinez Garcia J.C., Lattuada M. (2017). Reinterpretation of the mechanical reinforcement of polymer nanocomposites reinforced with cellulose nanorods. J. Appl. Polym. Sci..

[12] Trache D., Hussin M.H., Haafiz M.K.M., Thakur V.K. (2017). Recentprogressincellulose nanocrystals: Sources and production. Nanoscale.

[13] Li Y., Liu Y., Chen W., Wang Q., Liu Y., Li J., Yu H. (2016). Facile extraction of cellulose nanocrystals from wood using ethanol and peroxide solvothermal pretreatment followed by ultrasonic nanofibrillation. Green Chem..

[14] El Miri N., Abdelouahdi K., Barakat A., Zahouily M., Fihri A., Solhy A., El Achaby M. (2015). Bio-nanocomposite films reinforced with cellulose nanocrystals: Rheology of film-forming solutions, transparency, water vapor barrier and tensile properties of films. Carbohydr. Polym..

[15] Abdollahi M., Alboofetileh M., Rezaei M., Behrooz R. (2013). Comparing physico-mechanical and thermal properties of alginate nanocomposite films reinforced with organic and/or inorganic nanofillers. Food Hydrocoll..

[16] Wang L.-F., Shankar S., Rhim J.-W. (2017). Properties of alginate-based films reinforced with cellulose fibers and cellulose nanowhiskers isolated from mulberry pulp. Food Hydrocoll..

[17] Fatyeyeva K., Chappey C., Marais S. (2017). Biopolymer/clay nanocomposites as the high barrier packaging material: Recent advances. Food Packaging.

[18] Viseras C., Aguzzi C., Cerezo P., Bedmar M.C. (2008). Biopolymer–clay nanocomposites for controlled drug delivery. Mater. Sci. Technol..

[19] Qin Y., Wang W., Zhang H., Dai Y., Hou H., Dong H. (2018). Effects of Organic Modification of Montmorillonite on the Properties of Hydroxypropyl Di-Starch Phosphate Films Prepared by Extrusion Blowing. Materials.

[20] Chivrac F., Pollet E., Avérous L. (2009). Progress in nano-biocomposites based on polysaccharides and nanoclays. Mater. Sci. Eng. R Rep..

[21] Yufei C., Yang H., Qiwang D., Xiwang Z., Zhang Q. (2015). Preparation and properties of OMMT/PU composites. Adv. Mater. Sci. Eng..

[22] Luo Y., Liu H.Y., Zhang G.Z., Qu J.P. (2017). Morphological and thermal properties of PLA/OMMT. nanocomposites prepared via vane extruder. IOP Conf. Ser. Mater. Sci. Eng..

[23] Zakuwan S.Z., Ahmad I. (2018). Synergistic effect of hybridized cellulose nanocrystals and organically modified montmorillonite on κ-Carrageenan bionanocomposites. Nanomaterials.

[24] Kargarzadeh H., Johar N., Ahmad I. (2017). Starch biocomposite film reinforced by multiscale rice husk fiber. Compos. Sci. Technol..

[25] Zafar R., Zia K.M., Tabasum S., Jabeen F., Noreen A., Zuber M. (2016). Polysaccharide based bionanocomposites, properties and application: A review. Int. J. Biol. Macromol..

[26] Zubov A., Sin G. (2018). Multiscale modeling of poly (lactic acid) production: From reaction conditions to rheology of polymer melt. Chem. Eng. J..

[27] Noranizan I.A., Ahmad I. (2012). Effects of fiber loading and compatibilizer on rheological, mechanical and morphology behaviors. Open J. Polym Chem..

[28] Troedec M.L., Sedan D., Peyrotout C., Bonnet J.P., Smith A., Guinebretiere R., Gloaguen V., Krausz P. (2008). Influence of various chemical treatments on the composition and structure of hemp fibers. Compos. Part A Appl. Sci. Manuf..

[29] Kim J.S., Lee Y.Y., Kim T.H. (2016). A review on alkaline pretreatment technology for bioconversion of lignocellulosic biomass. Bioresour. Technol..

[30] El-Shekeil Y.A., Sapuan S.M., Khalina A., Zainuddin M., Al-Shuja’a E.S.O. (2012). Influence of chemical treatment on the tensile properties of jenaf fiber reinforced thermoplastic polyurethane composite. Express Polym. Lett..

[31] El Achaby M., Kassab Z., Barakat A., Aboulkas A. (2018). Alfa fibers as viable sustainable source for cellulose nanocrystals extraction: Application for improving the tensile properties of biopolymer nanocomposite films. Ind. Crop. Prod..

[32] Kargarzadeh H., Sheltami R.M., Ahmad I., Abdullah I., Dufresne A. (2015). Cellulose nanocrystal reinforced liquid natural rubber toughened unsaturated polyester: Effects of filler content and surface treatment on its morphological, thermal, mechanical, and viscoelastic properties. Polymer.

[33] Mariano M., Cercená R., Soldi V. (2016). Thermal characterization of cellulose nanocrystals isolated from sisal fibers using acid hydrolysis. Ind. Crop. Prod..

[34] Johar N., Ahmad I. (2012). Morphological, thermal, and mechanical properties of starch biocomposite films reinforced by cellulose nanocrystals from rice husks. BioResources.

[35] Zainuddin S.Y.Z., Ahmad I., Kargarzadeh H. (2013). Cassava starch biocomposites reinforced with cellulose nanocrystals from kenaf fibers. Compos. Interface.

[36] Li J., Cha R., Mou K., Zhao X., Long K., Luo H., Zhou F., Jiang X. (2018). Nanocellulose-based antibacterial materials. Adv. Healthc. Mater..

[37] Netoa W.P.F., Silverio H.A., Dantas N.O., Pasquini D. (2013). Extraction and characteristion of cellulose nanocrystals from agro-industrial residue- Soy hulls. Ind. Crop. Prod..

[38] Indicula M., Malhotra S.K., Joseph K., Thomas S. (2005). Dynamic mechanical analysis of randomly oriented intimately mixed short banana/sisal hybrid fiber reinforced polyester composites. Compos. Sci. Technol..

[39] Pan M.Z., Zhang S.Y., Zhou D.G. (2010). Preparation and properties of wheat straw fiber-polypropylene composites. Part II. Investigation of surface treatments on the thermo-mechanical and rheological properties of the composites. J. Compos. Mater..

[40] Tabatabaei S.H., Carreau P.J., Ajji A. (2009). Rheological and Thermal Properties of Blends of a Long-Chain Branched Polypropylene and Different Linear Polypropylenes. Chem. Eng. Sci..

[41] Zhou C., Wang Q., Wu Q. (2012). UV-initiated crosslinking of electrospun poly (ethyleneoxide) nanofibers with pentaerythritoltriacrylate: Effect of irradiation time and incorporated cellulose nanocrystals. Carbohydr. Polym..

[42] Zarina S., Ahmad I. (2015). Biodegradable Composite films based on κ-carrageenan reinforced by cellulose nanocrystals form kenaf fibers. BioResources.

[43] Martins J.T., Cerqueira M.A., Bourbon A.I., Pinheiro A.C., Souza B.W.S., Vicente A.A. (2012). Synergistic effects between k-carrageenan and locust bean gum on physicochemical properties of edible films made thereof. Food Hydrocoll..

[44] Yadav M., Chiu F.-C. (2019). Cellulose nanocrystals reinforced k-carrageenan based UV resistant transparent bionanocomposite films for sustainable packaging applications. Carbohydr. Polym..

[45] Roy S., Rhim J.-W. (2019). Preparation of carrageenan-based functional nanocomposite films incorporated with melanin nanoparticles. Coll. Surf. B.

[46] Chen J., Chen W., Duan F., Tanga Q., Li X., Zeng L., Zhang J., Xing Z., Dong Y., Jia L. (2019). The synergistic gelation of okra polysaccharides with kappa-carrageenan and its influence on gel rheology, texture behaviour and microstructures. Food Hydrocoll..

[47] Abdorreza M.N., Cheng L.H., Karim A.A. (2011). Effects of plasticizers on thermal properties and heat seability of sago starch films. Food Hydrocoll..

